# Multi-trophic communities re-establish with canopy cover and microclimate in a subtropical forest biodiversity experiment

**DOI:** 10.1007/s00442-021-04921-y

**Published:** 2021-04-25

**Authors:** Felix Fornoff, Michael Staab, Chao-Dong Zhu, Alexandra-Maria Klein

**Affiliations:** 1 Chair of Nature Conservation and Landscape Ecology, Faculty of Environment and Natural Resources, University of Freiburg, Tennenbacherstraße 4, 79106 Freiburg, Germany; 2 Ecological Networks, Technical University of Darmstadt, Schnittspahnstraße 3, 64287 Darmstadt, Germany; 3 Key Laboratory of Zoological Systematics and Evolution, Institute of Zoology, Chinese Academy of Sciences, 1 Beichen West Road, Chaoyang District, Beijing, 100101 People’s Republic of China; 4 College of Biological Sciences, University of Chinese Academy of Sciences, No.19(A) Yuquan Road, Shijingshan District, Beijing, 100049 People’s Republic of China

**Keywords:** Bees, Community assembly, Hymenoptera, Trees, Trophic interactions

## Abstract

Plant diversity affects multi-trophic communities, but in young regrowth forests, where forest insects are in the process of re-establishment, other biotic and also abiotic factors might be more important. We studied cavity-nesting bees, wasps and their natural enemies along an experimental tree diversity gradient in subtropical South-East China. We compared insect communities of experimental young forests with communities of established natural forests nearby the experiment and tested for direct and indirect effects of tree diversity, tree basal area (a proxy of tree biomass), canopy cover and microclimate on bee and wasp community composition, abundance and species richness. Finally, we tested if the trophic levels of bees, herbivore-hunting wasps, spider-hunting wasps and their natural enemies respond similarly. Forest bee and wasp community composition re-established towards communities of the natural forest with increasing tree biomass and canopy cover. These factors directly and indirectly, via microclimatic conditions, increased the abundance of bees, wasps and their natural enemies. While bee and wasp species richness increased with abundance and both were not related to tree diversity, abundance increased directly with canopy cover, mediated by tree biomass. Abundance of natural enemies increased with host (bee and wasp) abundance irrespective of their trophic position. In conclusion, although maximizing tree diversity is an important goal of reforestation and forest conservation, rapid closure of canopies is also important for re-establishing communities of forest bees, wasps and their natural enemies.

**Supplementary Information:**

The online version contains supplementary material available at 10.1007/s00442-021-04921-y.

## Introduction

Forests harbor 80% of Earth’s biodiversity and provide crucial ecosystem functions, but are in constant decline through deforestation (FAO 2011; Basset et al. 2012). Afforestation is a way to counterbalance this negative trend on forest area (FAO 2011, 2016; Hansen et al. 2013) and secondary forests of different successional stages become increasingly important for the conservation of forest biodiversity and functions (Dunn 2004; Schowalter 2012; FAO 2016). Ecosystem multifunctionality and biodiversity are driven by trophic interactions and biodiversity at multiple trophic levels (Soliveres et al. 2016; Schuldt et al. 2018). To investigate interactions and diversity at different trophic levels, cavity-nesting insects are an established study system (Tscharntke et al. 1998; Staab et al. 2018). These communities consist of Hymenoptera and their natural enemies. They represent multiple trophic levels ranging from herbivores to parasitoids of predators and mediate functions such as pollination, herbivore-control and parasitism (Staab et al. 2018).

### Community re-establishment

After forest clearance, the regrowth is typically characterized by non-forest species, but forest insects naturally disperse from surrounding forests into new forest plantations and re-establish species-rich communities within the first years of forest growth (Hilt and Fiedler 2005; Yeeles et al. 2017; Araújo et al. 2018; Hethcoat et al. 2019). For example, comparing forest interiors to forest edges, or following forest thinning, removal and restoration, cavity-nesting bees, wasps and their natural enemies had comparable species numbers, but showed distinct community compositions (Oliveira et al. 2017; Iantas et al. 2017; da Rocha-Filho et al. 2017; Araújo et al. 2018; Nether et al. 2019). Within these and further studies, plant diversity, biomass, canopy area and microclimate are discussed as drivers of multi-trophic forest insect communities (Stangler et al. 2015; Mayr et al. 2020). To the best of our knowledge, cavity-nesting bees and wasps have not been studied in forest biodiversity ecosystem functioning (BEF) experiments. By comparing bee and wasp communities of young regrowth forests in a forest BEF-experiment to communities of a natural forest, we expect young forest communities to change with biotic and abiotic factors towards natural forest communities (hypothesis 1).

### Tree diversity, biomass and microclimate effects on bees and wasps

Biodiversity-ecosystem functioning relationships emphasize the importance of bottom-up effects of biodiversity for functioning within and across trophic levels (Tilman et al. 2001; Scherber et al. 2010; Allan et al. 2013; Fornoff et al. 2019; Schuldt et al. 2019). For example, while Mayr et al. (2020) found no effect of resource diversity on cavity-nesting bee and wasp richness, Ebeling et al. (2008) observed more flower-visiting bee species and Fabian et al. (2014) more cavity-nesting wasp species in more diverse plant communities. In the here studied BEF-experiment, Hemiptera species richness and abundance increased more than additive with local tree species richness and Hemiptera species used more diverse resources at high local tree species richness, which indicates that resource complementarity might be responsible for the biodiversity increase (Fornoff et al. 2019). Similarly, Zhang et al. (2017) showed that increasing tree species richness increased the abundance of generalist herbivores, which potentially provide more resources for predators. Hence, we expect abundance and species richness especially of the lower trophic levels (bees and herbivore-hunting wasps more directly depending on plants as food resources) to benefit from tree species richness (hypothesis 2).

As part of the bottom-up control of higher trophic levels, theory and experiments support that with increasing resource availability larger and more diverse insect communities establish (Hunter and Price 1992; Siemann 1998; Srivastava and Lawton 1998). Bees and wasps might benefit from more abundant flowers (Ebeling et al. 2011), more herbivores (Zhang et al. 2017; Schuldt et al. 2019), or more shelter provided by increasing plant biomass in young forests. For early forest growth, the vegetation quantity hypothesis posits that increase in total plant biomass outweighs effects of qualitative plant community measures, such as complementarity, on ecosystem processes (Grime 1998; Lavorel et al. 2007; Lohbeck et al. 2015). Thus, we expect a direct effect of biomass on abundance and species richness of herbivores (bees) and herbivore-hunting wasps for young regrowth forest, as with biomass resource availability may increase (hypothesis 3).

Tree growth increases canopy cover and affects microclimatic conditions through increased evapotranspiration and decreased sun exposure below the canopy (Messier et al. 1998; Lebrija-Trejos et al. 2011). Cavity-nesting bees and wasps respond to microclimate and available food resources (Everaars et al. 2011; Ebeling et al. 2011; Mayr et al. 2020), and species may disperse several kilometers to reach nesting habitats with suitable microclimate (Bommarco et al. 2010; Everaars et al. 2011; Stangler et al. 2015). In this context, the field of dreams hypothesis posits that through the establishment of the fundamental habitat characteristics the species typical for the habitat type should recolonize (Palmer et al. 1997). This hypothesis was originally formulated for wetland restoration where with re-establishing the right soil water levels species are expected to recolonize. The same could be true for afforestation when trees are planted or left to natural regeneration, but no reintroduction of associated forest insects is accomplished. Therefore, we expect increasing abundance and species richness of forest bees and wasps with increasing canopy cover and the subsequent establishment of fitting abiotic forest characteristics such as temperature and humidity (hypothesis 4).

### Top–down or bottom–up control

Bottom–up effects of species richness (e.g. complementarity) and biomass (e.g. productivity) should ascend through trophic levels, but weaken with increasing trophic level (Haddad et al. 2009; Scherber et al. 2010). Alternative to ascending bottom**–**up effects, the enemies’ hypothesis states that plant diversity can affect enemies directly and independent of intermediate trophic levels and enemies may express top–down control on lower trophic levels (Root 1973; Staab et al. 2016; Staab and Schuldt 2020). For example, in (sub) tropical forests diverse mixtures are more likely to include trees with extra floral nectaries which were reported to support parasitoid insects (Heil 2015). Therefore, changes in plant species richness may simultaneously cause bottom–up and top–down effects and provide evidence of community control by both entangled effect directions (Hunter and Price 1992). In cavity-nesting insect communities, host and natural enemies are sampled in interaction. Using path analysis, we expect stronger correlations of natural enemies to the intermediate level of hosts than to the lowest (tree) level (hypothesis 5).

To inform forest management, we studied the relative importance of bottom-up and top-down effects induced by tree species richness, biomass and microclimate (which are all interrelated) for forest community re-establishment, abundance and species richness of multiple trophic levels in an experimental young regrowth forests and in comparison to a nearby established natural forest.

## Methods

### Experimental re-growth study sites and the established natural forest site

The study sites were established and maintained within the framework of the BEF-China experiment (Bruelheide et al. 2014), located in subtropical South-East China. The main data were taken at the Main Experiment study sites located near Xingangshan/Jiangxi Province (117° 54′ E, 29° 07′ N). The surrounding landscape (> 50 km radius) is a patchwork of small agricultural fields in the valleys and extensive, connected forest areas with conifer plantations or diverse secondary forests on the slopes. Local natural forests consist of 50% evergreen and 50% deciduous tree species. For detailed descriptions of the region and forest tree compositions see Bruelheide et al. (2011). The BEF-China Main Experiment is replicated at two study sites (site A and site B, Supplementary Fig. 1), 4 km apart from each other with a total area of 50 ha which makes it the globally largest tree diversity experiment (Bruelheide et al. 2014). For experimental forest establishment, previously existing secondary forest and tree plantations were harvested.

In total, 566 study plots of 25.8 × 25.8 m each in orthogonal projection were planted with 400 tree seedlings in a 20 × 20 grid system with 1.29 m distance between planted seedlings. At each study site, a tree diversity gradient of 24 tree species was planted (1, 2, 4, 8, 16, 24 species), obtained from a total species pool of 40 tree species in 2009 (site A) and 2010 (site B). Both study sites share eight tree species and have 16 site-specific tree species. All trees are represented in monocultures and all higher diversity mixtures represent randomly assembled species richness mixtures. For a comprehensive description of the BEF-China Experiment, we refer to Yang et al. (2013) and Bruelheide et al. (2014). To allow comparison to a natural forest embedded within the same landscape, 27 Comparative Study Plots (CSPs) 25 km away Gutianshan National Nature Reserve (GNNR; 29° 08′–29° 17′ N, 118° 02′–118° 11′ E) were established. Plots measured 30 × 30 m and included forest ages of less than 20 to over 80 years since last land use (Bruelheide et al. 2011; Staab et al. 2014).

### Cavity-nesting hymenoptera communities

Trap nests offer nesting resources for cavity-nesting bees and wasps that reproduce, provision their offspring and develop in the pre-existing cavities (Krombein 1967; Tscharntke et al. 1998). Therefore, trap nests assess true resident species independent of activity patterns. The required proximity among nesting cavities, larval and adult food resources and suitable microclimatic conditions contributes to the species’ sensitivity to local habitat changes (Tscharntke et al. 1998; Stangler et al. 2015; Staab et al. 2018). The number of nests established in a trap nest quantitatively represents their permanent presence, excluding only visitors to the area (Ebeling et al. 2011; Fabian et al. 2014). The offspring of the distinct trophic levels of bees and wasps are attacked by a variety of parasitoid or predatory insect species, which allows for the simultaneous assessment of multiple trophic levels, which are either co-occurring or directly interacting. For assessing cavity-nesting insect responses to a local habitat, connectivity and short distances between trap nests can be beneficial. This is because individuals can choose their most suitable nesting site based on external factors surrounding the trap nest, while sampling takes place from the same pool of individuals. As all plots within each study site were located within the flight range of the sampled bees and wasps, the impact of edge effects and factors outside the studied plots were reduced. Therefore, plot effects are mainly driving the sampling (Ebeling et al. 2011; Fabian et al. 2014).

### Sampling design

At each study site (A and B), eight plots of each tree species richness level (1, 2, 4, 8, 16) were randomly selected. Since only two plots of the tree species richness level 24 at each site were established, we included them within the 16 tree species richness mixtures (see also Fornoff et al. 2019). At site B, all 16 site-specific monocultures were included. Therefore, the sampling resulted in 40 plots (five tree species richness levels x eight plots) at site A and in 48 plots (five tree species richness levels x eight plots + eight additional monocultures) at site B, see Supplementary Table 1 and Supplementary Fig. 1. Per plot, two poles with trap nest were installed 11 m apart from each other (along a SW–NE diagonal at the center of each plot) and 9 m away from the nearest adjacent plots (Ebeling et al. 2011). At a height of 1.3 m above ground, each pole was equipped with two trap nests at site A and with four trap nests at site B. The difference between numbers of traps was accounted for by including site as a predictor in the analyses. Each trap nest consisted of a PVC tube (22 cm × 10 cm, length × diameter) filled with 75 ± 9 (SD) Giant cane (*Arundo donax*) internodes of 20 cm length and diameters varying between 1 and 20 mm (Staab et al. 2014). Therefore, each trap nest provided approximately 150 cavities for Hymenoptera nest establishment. Traps were exposed in August 2014 and monthly checked for nests of bees and wasps until August 2015. Collected nests were directly replaced by internodes of the same diameter and reared in glass test tubes in the laboratory under ambient conditions. Hatched hosts (bees and wasps that constructed nests) and natural enemies (insects that parasitized or fed on host individuals, including kleptoparasites; jointly referred to as natural enemies for simplicity) were identified based on voucher specimens for each nest. All specimens were identified to species or morphospecies (full species list in Supplementary Table 2) by respective taxonomists, see acknowledgements. To compare Hymenoptera communities to the reference expected in a natural forest, the same sampling method, sampling duration and taxonomic approach, was applied in the 27 CSPs in the GNNR from September 2011 to October 2012. A detailed description for data acquisition at the CSPs can be found in Staab et al. (2014).

### Habitat measures at the plot level

Tree basal area, a measure of biomass per area, was calculated based on the basal diameter of living tree individuals of planted trees in 2015. Diameter measurements were taken from 36 central tree individuals in 1 and 2 tree species richness level plots and from the central 144 trees in 4, 8, 16 and 24 species richness level plots to include all planted tree species under each species richness treatment. Measurement took place 5 cm above ground to include all individuals (Huang et al. 2018). Basal area per tree was calculated from basal diameters using the circular function *A* = pi·*r*^2^. To account for the size of the plot (665.64 m^2^) and the area in which the trees were measured, basal area was calculated as m^2^ ha^−1^. Trees on site B were generally smaller as the site was planted one year after site A; however, the basal diameter ranges overlapped (mean m^2^ ha^−1^ ± SD: site A 12 ± 7, site B 7 ± 6). Above each trap nest, we measured canopy cover, which influences the microclimate and insolation, through hemispherical pictures taken at 1.3 m above ground (i.e. trap nest height) with a 140-mm fish eye lens, in October 2015. Canopy cover was calculated as percentage of black area of total image size using image J (www.imagej.net; see Supplementary Data 1) and the mean of both values per plot was used for data analysis. In a subset of 55 plots, including all tree species richness levels, relative humidity and temperature were measured hourly at plot centers using HOBO data loggers for the full sampling time of trap nests. Mean relative humidity and temperature were used for analyses. The data distribution and spearmen’s correlation coefficients between all predictors are shown in Supplementary Fig. 2.

### Data management

Only brood cells from which bee, wasp or natural enemy individuals hatched were considered, while other brood cells, e.g. when larvae had died prematurely from unknown causes, were excluded from analyses since morphological species identification was often impossible. For all statistical analyses, each plot of the experimental study sites and of the established natural forest was treated as one replicate. Therefore, all insect samples of one plot were pooled, combining monthly samples of all trap nests of both poles. The analysis follows two levels of detail: the first considers all nest-building bees and wasps as one community and all natural enemies as a second community to test general responses of community composition, species richness and abundance (number of constructed or attacked brood cells). The second more detailed level of the analysis accounts for the different habitat requirements and life histories of bee and wasp species, which might identify different responses of species richness and abundance. For this, the nest-building bee and wasp community was separated into three trophic levels based on their food resources. We assorted plants at the first trophic level, followed by bees as herbivores feeding on pollen and nectar of plants at the second trophic level. Herbivore-hunting wasps including not only all Vespidae feeding on caterpillars, but also aphid-hunting Pemphredonidae and katydid and caterpillar-hunting Sphecidae, were assorted at the third trophic level. Spider-hunting wasps (Pompilidae, few Sphecidae and some Crabronidae) provisioning their larva with spiders were grouped at the fourth trophic level, although it needs to be mentioned that spiders may feed on invertebrates of various trophic levels. The natural enemies were assigned one trophic level higher than their hosts, i.e. at the 3rd level for bee enemies, the 4th level for herbivore-hunting wasp enemies and at the 5th level for spider-hunting wasp enemies. Each enemy individual was classified based on the host nest it attacked (see Supplementary Fig. 3). Species richness and abundance for each host and natural enemy group was calculated per plot.

### Statistical analysis

All analyses were conducted in R 3.6.1 (www.r-project.org). To test if community composition of bees and wasps and natural enemies changes with tree species richness, basal area and canopy cover, we used non-metric multidimensional scaling (NMDS) to calculate community dissimilarity and performed correlation tests. Community dissimilarity was calculated as Morisita–Horn distance matrix with 1000 random starts to find the most stable solution using the ‘metaMDS’ function of the R package ‘vegan’ (Oksanen et al. 2017). This index is insensitive to undersampling and attributes lower weightings to rare species (Wolda 1981; Chao et al. 2006). Therefore, it emphasizes more on the effects of dominant and, hence, likely functionally important species. The variables basal area, canopy cover and tree species richness were tested for correlation with NMDS axis scores by permutation (*N* = 1000), using the ‘envfit’ function of the R package ‘vegan’ (Oksanen et al. 2017). Potential influence of study site (site A and B) was accounted for by introducing site as a blocking variable using the ‘strata’ argument in ‘envfit’. The natural forest plots showed higher values in tree species richness and basal area than the experimental regrowth plots. Therefore, they were only included to calculate the distance matrix and visualize the location of reference communities within the NMDS. All statistical tests were conducted excluding the natural forest (except permutation test of the natural forest community dissimilarity, see Supplementary Material), as the effect of the predictor variables would not be distinguishable from confounding factors such as location or year of sampling.

To explore how tree species richness directly or indirectly, via basal area or canopy cover, affected bee and wasp species richness and abundance (all bees and wasps combined, or separated into trophic levels) and if natural enemies responded in a similar way or directly to their hosts, we used structural equation models (SEM; Lefcheck 2016). To account for potential differences between the two experimental regrowth study sites (e.g. number of trap nests, years since planting, tree species composition and unknown environmental factors), we used site as a predictor in all SEM models.

The data of all bees and wasps and natural enemies were analyzed on two separate but nested datasets, as tree basal area and canopy cover were assessed in all 88 sampled plots, while relative humidity and temperature were only available from 55 plots. Thus, we tested one SEM excluding microclimate data but including all plots with insect observations and one SEM including microclimate data but only a subset of plots (see Supplementary Table 1). Additionally, one SEM for each host group of bees, herbivore-hunting wasps and spider-hunting wasps was calculated including all sampled plots and the corresponding natural enemies.

All SEMs followed the same bottom-up structure, with tree species richness at the bottom, basal area and canopy cover as the first responses, bee and was abundance and species richness as the second responses and natural enemy abundance and species richness as the top responses. An example ‘full’ SEM is provided as Supplementary Fig. 4. Only in the subset-SEM temperature and humidity were used as intermediate responses between basal area and canopy cover and bee and wasp abundance and species richness. All paths from bottom to the top and left to right (basal area to canopy cover and abundance to species richness) were tested. Each SEM was calculated using component models of which each included one response variable and all downstream responses and predictors as explanatory variables and study site as a co-variable in the ‘picewiseSEM’ package (Lefcheck 2016). Individual component models were Poisson generalized linear (responses: species richness and abundance) and Binomial generalized linear (response: canopy area) or linear (basal area) regression models. Model fit of each component model was evaluated using the DHARMa package (Hartig 2018) and in the case of overdispersion of Poisson models, a negative binomial distribution was used (MASS package; Ripley et al. 2018). The full SEM was backward-selected to achieve parsimony using AICc (Lefcheck 2016). Based on overall model fit calculated by Fishers’ *C* statistic, we evaluated whether any paths were missing from the model. Here a minimum AICc and a non-significant Fishers’ *C* indicate a best-fitting model balancing parsimony and necessity of parameters (Lefcheck 2016). When ΔAICc of two models with the same number of parameters was < 3 in model selection, the results of both alternative models were considered. All numeric predictor variables (log_2_ tree species richness and basal area) were scaled by subtraction of their mean values and division by their standard errors; binomial (canopy cover) and count (abundance and species richness of hosts and natural enemies) data were not scaled to preserve their error structure in the models.

## Results

The 544 trap nests providing in total approximately 81,600 trap cavities for 1 year yielded 3694 (1838 at site A and 1856 at site B) nests of bees and wasps. From these nests, 7146 brood cells were reared to insect imagines belonging to 93 species. The bee and wasp community consisted of 11 bee (Megachilidae, Colletidae) and 29 wasp species (Crabronidae, Vespidae, Pemphredonidae, Pompilidae, Sphecidae) with 2364 and 3132 individuals respectively. The natural enemy community reared from 1650 brood cells consisted of seven cuckoo-bee (Megachilidae), 33 wasp (Chrysididae, Ichneumonidae, Braconidae, Chalcidoidea), nine fly (Tachinidae, Drosophilidae, Sarcophagidae), three beetle (Meloidae, Dermastidae, Ripiphoridae) and one Strepsiptera (Stylopidae) species. The reference communities additionally contained one further cuckoo-bee, ten further wasp and 14 additional natural enemy species (see Supplementary Table 2 for a complete list). Enemy infestation rate was 14% for bees and 29% for wasps.

**Fig. 1:**
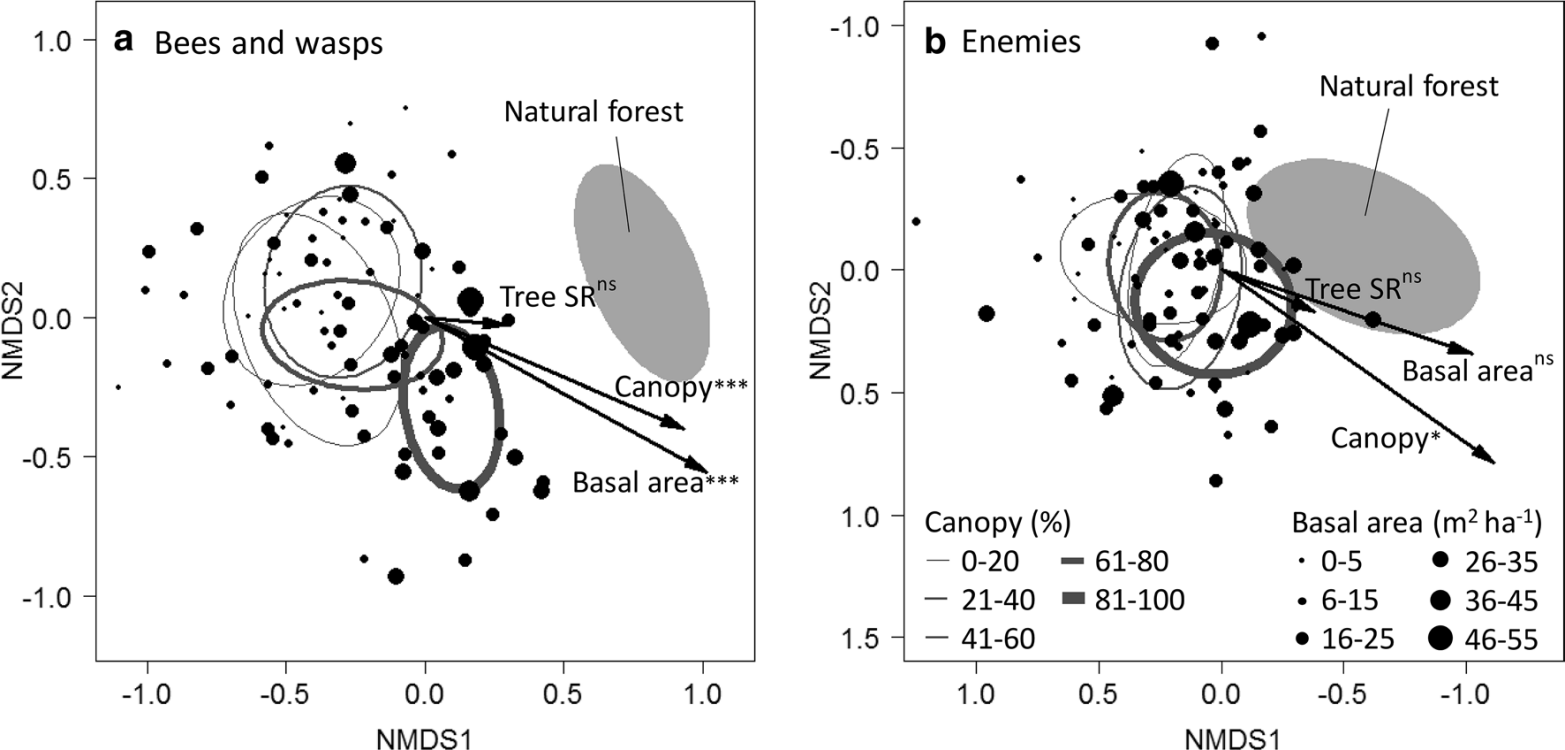
Non-metric multidimensional scaling (NMDS) showing the first two dimensions of ordinations of the cavity-nesting species community matrix for **a** bee and wasp species (stress = 0.190, *k* = 3) and **b** natural enemy (Enemies) species (stress = 0.189, *k* = 3). Ellipses show 95% confidence intervals of communities grouped by canopy cover (Canopy), with increasing line thickness of ellipses indicating increasing canopy cover. Points represent relative positions of communities and point size increases with basal area measured at the plots. The bee and wasp community changed with basal area and canopy cover, the enemy community changed with canopy cover. The filled ellipse represents the ‘reference communities’ of a nearby natural forest which were not used for environmental variable fitting. Black arrows indicate the post-hoc correlations with canopy cover, basal area and tree species richness (***P < 0.001, *P < 0.05, *n.s.* not significant). Similar results were obtained for communities separated into trophic groups, see Supplementary Fig. 5

Tree species richness had no significant effect on the cavity-nesting communities (Fig. 1). Bee and wasp community composition changed significantly with increasing canopy cover (*r*^2^ = 0.384, *P* < 0.0001) and basal area (*r*^2^ = 0.325, *P* < 0.0001; Fig. 1a). The position of the bee and wasp communities of the natural forest (gray ellipse, marking the 95% confidence interval (CI), in Fig. 1a) aligned approximately with increasing basal area and canopy cover. The distance between the ellipse of the natural forest communities, and the ellipses of the young forest communities grouped by canopy cover decreased with increasing canopy cover (Fig. 1a). The natural enemy community changed significantly with canopy cover (*r*^2^ = 0.103, *P* = 0.0276), but not with tree species richness or basal area. The 95% CI ellipse of the natural enemy communities of the natural forest was overlapping with the communities found at the young forest sites (Fig. 1b). Bee and wasp communities, separated into trophic groups, showed the same correlations with canopy area and basal area (Supplementary Fig. 5). Within the established natural forests, bee and wasp community composition was not correlated with stand age (Supplementary Fig. 6).

**Fig. 2:**
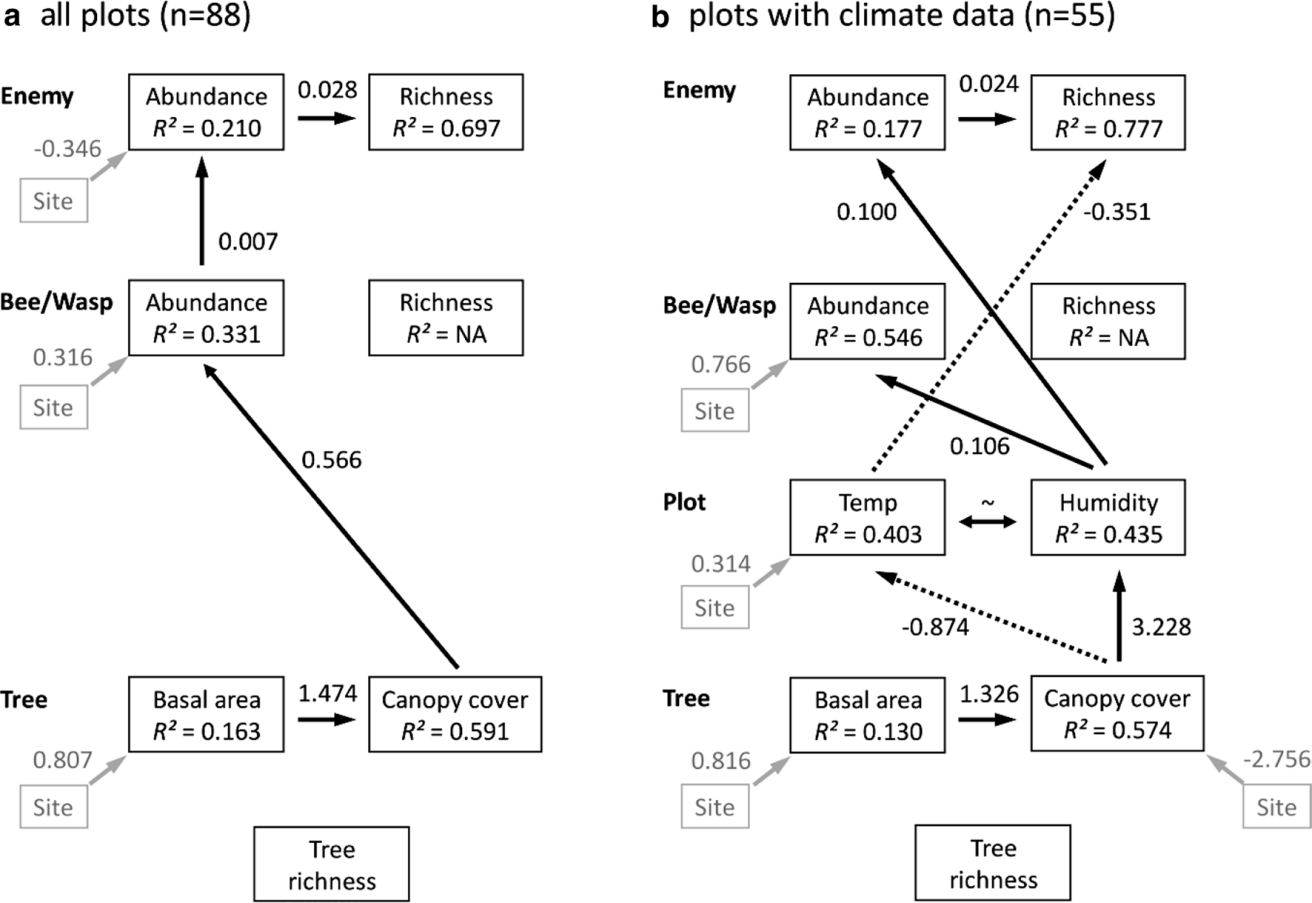
Final, most-parsimonious path models (paths significant at *P* < 0.05 shown) of young regrowth forests **a** all study plots and **b** study plots with microclimate data. Direct and indirect effects of tree species richness (tree richness; log2 of planted tree species richness) via tree basal area and canopy cover to species richness and abundance of bees and wasps and their natural enemies (Enemy) are illustrated, see Supplementary Fig. 4 for a ‘full’ model. All paths from bottom to the top and left to right were tested; solid black arrows indicate significant positive and dotted arrows negative paths. Unstandardized correlation coefficients are shown next to arrows. The effect of the covariate ‘site’ was tested on each response and is shown as the effect of the experimental young forest ‘site A’ (in comparison to ‘site B’), in gray arrows, if significant. The amount of variance explained by the model is given as coefficient of determination (*R*^2^). Paths are interdependent (~), absent paths are validated by non-significant difference of Fisher’s *C* compared to a *χ*^2^ distribution (all plots: *n* = 88, Fisher’s *C* = 36.826, *df* = 34, *P* = 0.339, plots with climate data: *n* = 75, Fisher’s *C* = 52.325, *df* = 55, *P* = 0.182). A statistical alternative model for the model including climate data replaces the negative correlation between canopy cover and temperature with a negative correlation between basal area and temperature (ΔAICc = 1, see Supplementary Table 3)

The SEM including all cavity-nesting bees and wasps showed that tree species richness had no direct or indirect effect on bees and wasps (Fig. 2a, Supplementary Table 3). Canopy cover increased with basal area (estimate = 1.47 ± 0.34 SE, *df* = 86, *P* < 0.0001), while, in turn, bee and wasp abundance increased with canopy cover (estimate = 0.56 ± 0.18 SE, *df* = 85, *P* = 0.0022). Likewise, with increasing bee and wasp abundance natural enemy abundance increased (estimate = 0.007 ± 0.002 SE, *df* = 85, *P* < 0.0001) and with abundance their species richness (estimate = 0.028 ± 0.004 SE, *df* = 86, *P* < 0.0001). Furthermore, the SEM with temperature and humidity showed that the effect of canopy cover was mediated through temperature, which decreased natural enemy species richness (estimate = -0.351 ± 0.125 SE, *df* = 52, *P* = 0.0051), and relative humidity, which increased host (estimate = 0.106 ± 0.031 SE, *df* = 52, *P* = 0.0006) and natural enemy abundance (estimate = 0.099 ± 0.036 SE, *df* = 53, *P* = 0.0060, Fig. 2 B, Supplementary Table 3). An alternative statistical model replaced the correlation between canopy cover and temperature with a correlation between basal area and temperature, while all other correlations remained constant (ΔAICc = 1, see Supplementary Table 3).

**Fig. 3:**
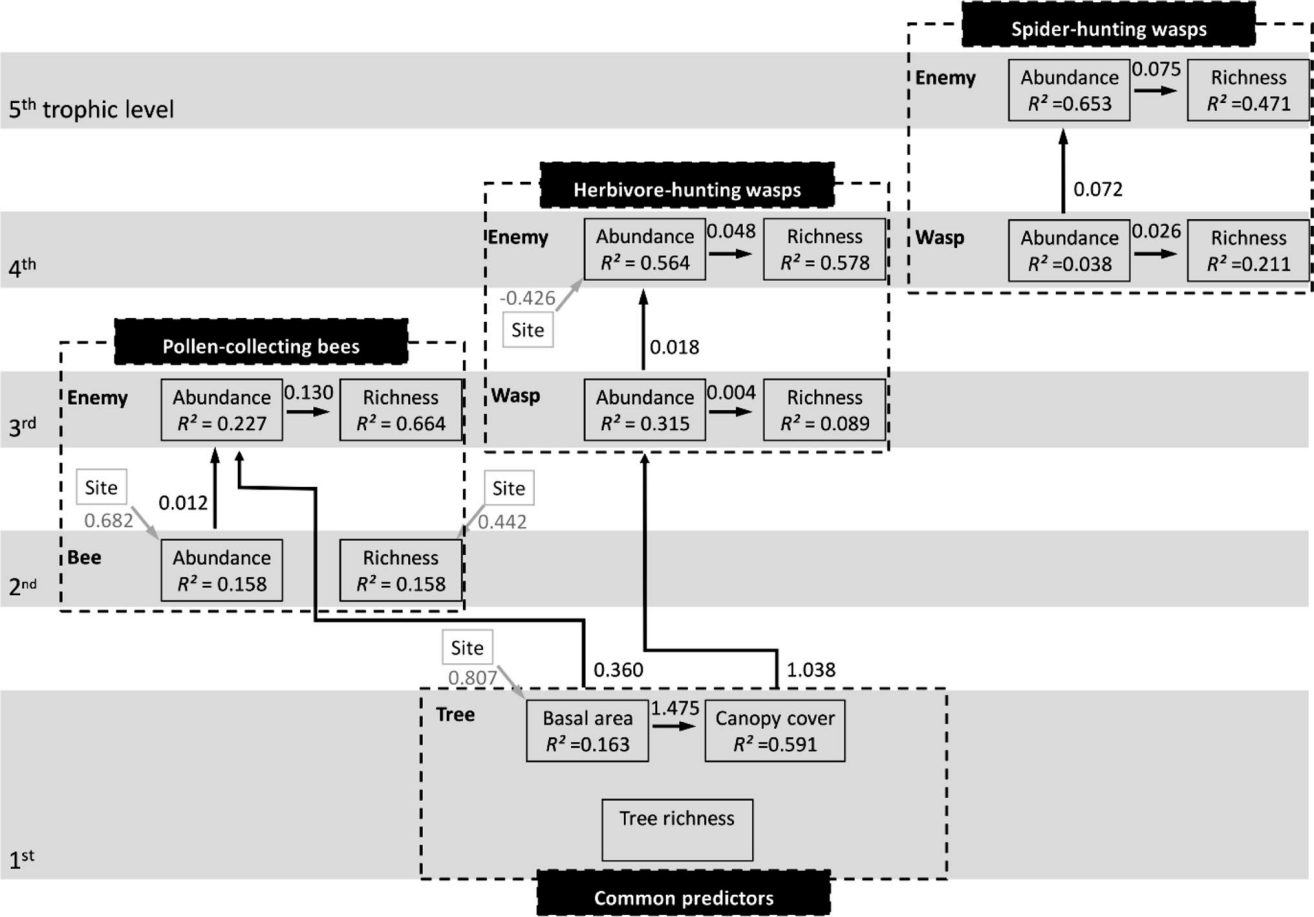
Results of separate SEMs for pollen-collecting bees, herbivore-hunting and spider-hunting wasps and their natural enemies, depicted in accordance with their trophic position (Supplementary Table 3). Black arrows show significant paths (*P* < 0.05) of direct and indirect effects of tree species richness (SR; log2 of planted tree species richness) via tree basal area and canopy cover to the species richness and abundance of bees, wasps and their natural enemies (Enemy) (Supplementary Table 3). Within each SEM, tree species richness, basal area and canopy cover (‘Common predictors’—of all SEMs) were included and all paths from bottom to top and from left to right tested. Unstandardized correlation coefficients are shown next to arrows. The effect of covariate ‘site’ was tested on each response; significant effects of the experimental young forest ‘site A’ in comparison to ‘site B’ are shown, as gray arrows. The amount of variance explained by the model is given as coefficient of determination (*R*^2^). Absent paths are validated by non-significant difference of Fisher’s *C* compared to a *χ*^2^ distribution (herbivore-hunting wasps: *n* = 88, Fisher’s *C* = 28.933, *df* = 28, *P* = 0.416, bees: *n* = 88, Fisher’s *C* = 19.708, *df* = 32, *P* = 0.956, spider-hunting wasps: *n* = 88, Fisher’s *C* = 22.629, *df* = 32, *P* = 0.89). A statistical alternative model for herbivore-hunting wasps replaces the correlation between canopy and wasp abundance with a correlation of basal area and wasp abundance (ΔAICc = 1, see Supplementary Table 3)

When separating the cavity-nesting community into their respective trophic levels (Fig. 3, Supplementary Table 3), SEMs showed that cavity-nesting bees (2nd level) were not related to tree (1st level) factors, while bee enemy (3rd level) abundance directly increased with basal area (estimate = 0.356 ± 0.132 SE, *df* = 78, *P* = 0.0070) and bee abundance (estimate = 0.012 ± 0.005 SE, *df* = 78, *P* = 0.0104). Herbivore-hunting wasp (3rd level) abundance increased with canopy cover (estimate = 1.038 ± 0.220 SE, *df* = 86, *P* < 0.0001) or alternatively with basal area (ΔAICc = 1, see Supplementary Table 3). Herbivore-hunting wasp enemy (4th level) abundance increased with their host abundance (estimate = 0.0476 ± 0.0073 SE, *df* = 83, *P* < 0.0001) and enemy species richness with enemy abundance (estimate = 0.0179 ± 0.0022 SE, *df* = 82, *P* < 0.0001). In contrast, spider-hunting wasps (4th level) and their enemies (5th level), representing the highest trophic level of the cavity-nesting insects, were not related to any tree factor. There, species richness of spider-hunting wasps (estimate = 0.0258 ± 0.007 SE, *df* = 86, *P* < 0.0001) and their natural enemies (estimate = 0.075 ± 0.014 SE, *df* = 71, *P* < 0.0001) increased with their respective abundance (Fig. 3, Supplementary Table 3) and enemy abundance with host abundance (estimate = 0.072 ± 0.009 SE, *df* = 71, *P* < 0.0001).

## Discussion

In young regrowth forests, cavity-nesting bee and wasp community composition re-established with increasing canopy cover and basal area. Natural enemy abundance increased with bee and wasp abundance and microclimate. Bee and wasp abundance changed with microclimate, modulated by canopy cover and especially herbivore-hunting wasp abundance increased with increasing canopy cover. Canopy cover increased with tree basal area, which was shown to increase in the BEF-China Experiment with tree species richness by Huang et al. (2018). Therefore, biotic and abiotic factors connect young regrowth forest trees with multi-trophic interactions. These ascending effects indicate that bottom-up mechanisms likely contributed, but canopy cover and microclimate were the direct drivers of suitable habitats for forest characteristic cavity-nesting bee and wasp (host) species in the studied young subtropical forests.

### Forest characteristic multi-trophic bee and wasp community re-establishment

In young regrowth forests, communities of bees and wasps changed with increasing canopy cover. It is known that cavity-nesting Hymenoptera species compositions are distinct for forest interiors and forest edges (Oliveira et al. 2017; da Rocha-Filho et al. 2017) and change with forest modification (Iantas et al. 2017; Nether et al. 2019). Likewise, young forests can be expected to harbor different communities, sometimes even with more species compared to old-natural forests (Yeeles et al. 2017; Araújo et al. 2018). We found more insect species in young regrowth forest plots which was possibly related to lower sampling intensity at the natural forest plots, or inter-annual fluctuations of insect populations, or potential higher habitat heterogeneity of the experimental sites. Although we cannot exclude that this might have affected our comparison, young and natural forest plots had shared and exclusive species, indicating a joint species pool. Communities of the three trophic groups also individually responded to basal area and canopy cover. Therefore, niches of species within trophic groups are distinct. For example, in this study, *Megachile spissula* is a flower generalist that likely benefits from herbal flower abundance, but others might be specialized on a specific tree species. The herbivore-hunting *Isodontia nigella* preys on katydids and constructs nest using grass, while *Allorhynchium chinense* exploits caterpillars and uses resin for nest construction, resources potentially more abundant in closed canopy habitat. The spider-hunting *Deuteragenia ossarium* was observed most in the natural forests and *Sceliphron deforme* was only present at the young forest sites, both differ in size and likely have different preferred prey species and habitat niches. A surrogate factor for a range of niche components, affecting for example nesting and food resources and microclimate that explained community differences, could be canopy cover. The prevailing influence of canopy cover, as potential fundamental habitat characteristic, on establishing forest communities is in support of the field of dreams hypothesis (Palmer et al. 1997), and implies that for conserving forest insects rapid canopy closure seems important to re-establish habitat characteristics typical of natural forests.

In contrast, the vegetation quantity hypothesis received less support by our analyses (Lohbeck et al. 2015). Even though host communities changed with tree basal area (biomass), canopy cover explained more variance and was, therefore, more important for bee and wasp communities in the statistical analyses. Dependency on resources might be more important for antagonistic herbivores (Zhang et al. 2017; Fornoff et al. 2019) and less important for mutualistic herbivores such as bees (Mayr et al. 2020). However, in the studied young forest sites, biomass and canopy cover were correlated and the SEM analysis did not separate their effects on herbivore-hunting wasp abundance (Supplementary Fig. 2, Supplementary Table 3). Therefore, long-term studies, in which canopy cover saturates while biomass changes, are needed to investigate the whole process of re-establishment and its drivers.

In accordance with the host communities, enemy communities changed with canopy cover. As natural enemies are directly depending on the availability of their hosts (SEM, Staab et al. 2016), this response should be expected. However, the natural enemy communities of the young and the established natural forest were overlapping, indicating potential distinct responses of enemies to their environment. The SEM analyses showed that natural enemies responded not only to host abundance but also to humidity (all enemies) and tree basal area (bee enemies). Hence the plant level might directly influence enemy communities (Hranitz et al. 2009; Staab et al. 2016; Schuldt et al. 2019). Enemy-community change independent of host communities indicates potential top-down control of host communities (Staab and Schuldt 2020). However, host and enemy abundance increased in parallel and top-down effects were likely masked by this correlation and are, therefore, difficult to evaluate within the studied experimental set up. Together, our results suggest that for conserving natural enemies host abundance is required, but further habitat characteristics, such as microclimate, might also be important (Klein et al. 2006; Staab et al. 2016).

### Microclimate drives abundance of nesting bees and wasps and natural enemies

Microclimate, in this context used as the temperature and humidity regime measured at the center of an individual plot, was shaped by canopy cover, although temperature alternatively correlated with basal area. Humidity directly affected bee and wasp abundance. For cavity-nesting bees and wasps, microclimatic conditions affect the reproductive success, as after nest construction no further brood care behavior is conducted (Westrich 1996; Hranitz et al. 2009; Stangler et al. 2015). Humidity directly affects the probability of parasitoid and pathogen spread, dehydration and nest destruction (Westrich 1989, 1996; Hranitz et al. 2009). Although humidity was stronger correlated with bees and wasps than temperature in the SEM, humidity may act as a proxy for other microclimatic variables, for example minimum- or maximum-, spring- or winter-, humidity or temperature and their variability over time. It is known that temperature extremes can cause larval mortality or reduced growth, for example the heat-tolerant cavity-nesting *Megachile apicalis* seems to balance heat-induced mortality in sun exposed nesting sites against nesting at shaded sites with the compromise of higher parasitism rates (Hranitz et al. 2009; Radmacher and Strohm 2011). Hence, bees and wasps can be expected to choose nesting sites, in respect to their species-specific microclimatic habitat requirements and tolerances (Hranitz et al. 2009; Mayr et al. 2020).

Although, following this reasoning, all bees and wasps regardless of trophic level will have a preferred environmental and biological niche, the effects found in this study might be driven by herbivore-hunting wasps (Fig. 3). We assume that developing herbivore-hunting wasps might be more sensitive to extreme temperatures than developing bees in trap nests, as their food resource, paralyzed insects, might decay faster compared to bee resources, a mix of nectar and pollen. More intensive taxa/species-specific investigations are needed to either explain different responses of trophic groups or detect drivers of population and community changes of bees and spider-hunting wasps.

During the process of forest growth, total standing biomass and microclimatic conditions undergo profound changes (Swanson et al. 2011). We showed that tree biomass increases canopy cover which modified temperature and humidity. In line with this reasoning, our results showed an increase in bee and wasp abundance with canopy cover and an increase of bee and wasp and natural enemy abundance with humidity. We also showed a community shift with biomass and canopy cover towards natural forest communities. This indicates that in young regrowth forests, re-establishing forest-adapted insects select for their favorable microclimatic conditions. Therefore, the results suggest that restored abiotic habitat characteristics are fundamental for re-establishment of forest characteristic bee and wasp species (Palmer et al. 1997). To which extend tree identity could drive this relation should be addressed in further studies.

### Bottom–up effects across trophic levels

Diversity at lower trophic levels can increase diversity at higher trophic levels through a suite of mechanisms including resource complementarity and availability (e.g. Srivastava and Lawton 1998; Fornoff et al. 2019; Schuldt et al. 2019). Although we did not quantify resource availability for cavity-nesting bees and wasps, food resources, such as pollen for bees or herbivores for herbivore-hunting wasps, may generally be more abundant in diverse tree communities (Krombein 1967; Srivastava and Lawton 1998; Hector et al. 1999; Ebeling et al. 2008; Fornoff et al. 2017, 2019; Zhang et al. 2017). Likewise, host availability should increase natural enemy abundance. This bottom–up diversity–diversity relationship is expected to ascend through the trophic chain and weaken with increasing trophic distance between trophic levels (Scherber et al. 2010; Fornoff et al. 2019). Although Mayr et al. (2020) and we expected a positive response of bees to flower diversity, as was shown for flower-visiting bee richness and cavity-nesting bee abundance in grasslands (Ebeling et al. 2008, 2011) and other herbivores in in our experimental study sites (Zhang et al. 2017; Fornoff et al. 2019), cavity-nesting bees were not related to the plant level. Either bees are not bottom-up controlled in young forests or this might change once more trees start to flower. Future studies should manipulate the herb layer to better include the availability of flower resources within the study plots. In contrast to bees, bee enemies and herbivore-hunting wasps (both at the 3rd level) but not spider-hunting wasps (4th level) and their natural enemies (5th level) were affected by the tree level (1st). Additionally, the abundance of natural enemies was positively correlated to host abundance, hence directly linked trophic levels, irrespective of their trophic position (2nd–3rd, 3rd–4th, 4th–5th trophic level) pointing to bottom-up rather than to top down effects. Except for bees, direct bottom-up effects seem to decrease with increasing distance from trees or distance between tropic levels.

The BEF China Experiment manipulates the plant level, which enables the detection of bottom-up mechanisms only. However top-down forces related to the enemies hypothesis may have also impaired our results (Root 1973; Staab and Schuldt 2020). Bee enemy abundance was directly linked to tree basal area and overall enemy abundance responded to humidity irrespective of host abundance. Schuldt et al. (2019) showed that abundances of higher trophic levels were associated with factors of the tree level. This indicates an independent response of antagonists to changes at the tree level and hence potential tree diversity-driven top-down effects. However, for bees and both wasp groups (hosts) we found consistent positive correlations between hosts and natural enemies. Hosts of natural enemies were not accounted for in Schuldt et al. (2019), but provide a more direct connection to the plant level. Therefore, we cannot exclude top-down effects but show that bottom-up effects were stronger in the studied young experimental sites with a manipulated bottom level.

Taken together, bottom-up effects can be expected within multi-trophic communities, especially between directly dependent trophic levels in young forest. As bottom–up effects are known to strengthen over time (Tilman et al. 2006; Allan et al. 2013; Huang et al. 2018) and, for example, trees start to flower after a certain age, multi-trophic bee and wasp communities may increasingly respond to changes within the tree level. However, this dependence might be a characteristic of young forests and only be present for a certain time. In the natural forest, Hymenoptera abundance and community compositions were constant at increasing forest age (Staab et al. 2016; Araújo et al. 2018). Therefore, bottom-up effects might decline and top-down control increasingly control populations with further forest growth (Staab and Schuldt 2020).

We conclude that different drivers structure community composition, diversity and abundance of cavity-nesting bees and wasps and their natural enemies in young forests. The results of this study demonstrate the high re-establishment potential of forest Hymenoptera communities during forest succession, when natural forests are still in the vicinity, and the immediate response of cavity-nesting communities to small changes in their environment. The combined effect of tree diversity, which mediates productivity over time (Chen et al. 2018; Huang et al. 2018), and the shift of Hymenoptera communities with canopy cover towards communities found in natural forests, emphasize the value of young diverse regrowth forests to establish natural forest insect communities.

## Supplementary Information

Below is the link to the electronic supplementary material.

Supplementary file1 (DOCX 636 KB)

## Data Availability

All data are available at https://china.befdata.biow.uni-leipzig.de, for insects see data sets 486, 487, 564 and 565, for tree inventories, see data sets 593 and 594 and for microclimate, see data set 559. Data on canopy cover is available in the Supplementary Material.

## Code availability

No custom code was used.
