# Supplementary material for: Multi-trophic communities re-establish with canopy cover and microclimate in a subtropical forest biodiversity experiment
Source: Oecologia. 2021 Apr 25;196(1):289–301. doi: 10.1007/s00442-021-04921-y (PMC8139880; doi:10.1007/s00442-021-04921-y)
Supplement: Supplementary file 1 — Supplementary file1 (DOCX 636 KB) [file 442_2021_4921_MOESM1_ESM.docx]

**Multi-trophic communities re-establish with canopy cover and microclimate in a subtropical forest biodiversity experiment**

Felix Fornoff et al.

**Supplementary material**

Supplementary Table 1:

Cavity-nesting insect communities were assessed in all plots of the ‘full data’. Microclimate data on temperature and relative humidity were only available for a subset of these plots.

|  | Tree SR | 1 | 2 | 4 | 8 | 16 |
| --- | --- | --- | --- | --- | --- | --- |
| Full data | Site A | 8 | 8 | 8 | 8 | 8 |
|  | Site B | 16 | 8 | 8 | 8 | 8 |
| Subset data | Site A | 8 | 8 | 4 | 2 | 2 |
|  | Site B | 16 | 7 | 4 | 2 | 2 |

Supplementary Table 2:

Species list and occurrences of host and natural enemy species, at site A & B in 2014/2015 (A & B) and at the CSPs (natural forest plots) in 2011/2012. Species are ordered by decreasing occurrence, hosts additionally by their respective functional group (Bee, Herb.-hunt: herbivore-hunting wasp, Spid.-hunt: spider-hunting wasp).

| Fun. group | Host species | A | B | CSP |  | Natural enemy species | A | B | CSP |
| --- | --- | --- | --- | --- | --- | --- | --- | --- | --- |
| Bee | *Megachile spissula* | 470 | 216 |  |  | *Amobia* sp. 1 | 131 | 184 | 125 |
| Bee | *Heriades sauteri* | 301 | 300 |  |  | *Chrysis smaragdula* | 83 | 174 | 117 |
| Bee | *Osmia taurus* | 60 | 48 | 275 |  | Ichneumonidae sp. 3 | 111 | 150 |  |
| Bee | *Megachile sculpturalis* | 152 | 101 | 87 |  | *Melittobia* sp. 1 | 84 | 174 |  |
| Bee | *Hoplitis carinotarsa* | 133 | 114 | 29 |  | *Lycogaster violaceipennis* | 67 | 17 | 93 |
| Bee | *Megachile disjunctiformis* | 191 | 63 |  |  | *Apanteles* sp. 1 | 2 | 2 | 88 |
| Bee | *Megachile abluta* | 72 | 27 | 33 |  | *Primeuchroeus kansitakuanus* | 9 | 37 |  |
| Bee | *Megachile rixator* | 52 | 22 |  |  | Tachinidae sp. 3 |  |  | 40 |
| Bee | *Megachile monticola* | 23 |  | 17 |  | Ichneumonidae sp. 1 | 6 | 4 | 29 |
| Bee | *Trachusa* *staabi* | 14 |  | 11 |  | *Coelioxys brevicaudata* | 16 | 16 | 4 |
| Bee | *Hylaeus* sp. 1 | 5 |  |  |  | *Euaspis basalis* | 32 | 4 | 3 |
| Herb.-hunt. | *Anterhynchium flavomarginatum curvimaculatum* | 859 | 555 | 1079 |  | Braconidae sp. 1 | 17 | 14 | 4 |
| Herb.-hunt. | *Anterhynchium flavomarginatum flavomarginatum* |  |  | 328 |  | *Coelioxys* sp. 4 | 30 | 4 |  |
| Herb.-hunt. | *Orancistrocerus drewseni* | 156 | 31 | 13 |  | *Anthrax aygula* | 19 | 14 |  |
| Herb.-hunt. | *Hoplammophila aemulans* |  |  | 199 |  | *Melittobia* sp. 2 | 11 | 22 |  |
| Herb.-hunt. | *Epsilon fujianensis* | 94 | 14 | 24 |  | *Trichrysis cyanea* | 6 | 23 |  |
| Herb.-hunt. | *Polemistus* sp. 1 | 114 |  |  |  | *Euaspis* sp. 1 | 17 | 10 |  |
| Herb.-hunt. | *Allorhynchium chinense* | 47 | 63 | 1 |  | *Lycogaster nigralva* |  | 4 | 23 |
| Herb.-hunt. | *Isodontia nigella* | 29 | 73 |  |  | *Coelioxys fenestrata* | 18 | 2 |  |
| Herb.-hunt. | *Stenodynerus chinensis chinensis* | 11 | 90 |  |  | Ichneumonidae sp. 7 | 7 | 1 | 10 |
| Herb.-hunt. | *Subancistrocerus kankauensis* | 8 | 76 |  |  | Sarcophagidae sp. 4 | 4 | 14 |  |
| Herb.-hunt. | *Pareumenes quadrispinosus transitorus* | 9 | 36 |  |  | *Irenangulus* sp. 1 |  |  | 15 |
| Herb.-hunt. | *Euodynerus quadrifasciatus* | 8 | 30 |  |  | *Leucospis japonica* | 7 | 4 | 3 |
| Herb.-hunt. | *Eumenes quadratus urainus* | 5 | 17 |  |  | *Coelioxys ducalis* |  |  | 12 |
| Herb.-hunt. | *Discoelius wangi* | 10 | 11 | 1 |  | Ichneumonidae sp. 8 | 5 | 5 | 1 |
| Herb.-hunt. | *Ancistrocerus trifasciatus shibuyai* |  |  | 14 |  | *Melittobia clavicornis* | 8 | 2 |  |
| Herb.-hunt. | *Eumenes quadratus quadratus* |  | 11 | 2 |  | *Eurytoma* sp. 3 | 4 |  | 5 |
| Herb.-hunt. | *Ancistrocerus* sp. 1 | 8 | 1 |  |  | Diptera sp. 2 |  |  | 8 |
| Herb.-hunt. | *Rhopalum* sp. 1 | 8 | 1 |  |  | *Eurytoma* sp. 1 | 2 | 6 |  |
| Herb.-hunt. | *Pararrhynchium ornatum ornatum* |  | 3 |  |  | Sarcophagidae sp. 2 | 4 | 4 |  |
| Herb.-hunt. | *Ancistrocerus nigricornis* |  |  | 2 |  | Strepsiptera sp. 1 | 5 | 3 |  |
| Herb.-hunt. | *Anterhynchium* sp. 1 |  |  | 1 |  | *Zonitis japonica* |  | 1 | 7 |
| Herb.-hunt. | *Okinawepipona kogimai* |  | 1 |  |  | Ichneumonidae sp. 11 |  |  | 7 |
| Spid.-hunt. | *Auplopus carbonarius* | 209 | 248 |  |  | Drosophilidae sp. 1 |  |  | 6 |
| Spid.-hunt. | *Deuteragenia ossarium* | 16 | 9 | 213 |  | *Chrysis angolensis* |  | 5 |  |
| Spid.-hunt. | *Auplopus* sp. 4 |  |  | 75 |  | Dermestidae sp. 1 | 1 | 4 |  |
| Spid.-hunt. | *Deuteragenia* sp. 2 | 32 | 35 | 8 |  | Tachinidae sp. 4 |  |  | 5 |
| Spid.-hunt. | *Sceliphron deforme deforme* | 4 | 48 |  |  | Tachinidae sp. 6 |  |  | 4 |
| Spid.-hunt. | Pompilidae sp. 1 | 2 | 49 |  |  | Ichneumonidae sp. 4 |  |  | 4 |
| Spid.-hunt. | *Pison* sp. 1 | 30 | 10 |  |  | Ichneumonidae sp. 6 | 4 |  |  |
| Spid.-hunt. | *Trypoxylon* sp. 1 |  | 29 |  |  | Sarcophagidae sp. 1 | 1 | 3 |  |
| Spid.-hunt. | *Chalybion japonicum* |  | 9 | 5 |  | Diptera sp. 1 |  |  | 3 |
| Spid.-hunt. | *Deuteragenia* sp. 1 |  |  | 11 |  | *Apanteles* sp. 2 |  |  | 3 |
| Spid.-hunt. | *Auplopus* sp. 2 |  |  | 8 |  | Perilampidae |  | 3 |  |
| Spid.-hunt. | *Nitela* sp. 2 |  | 7 |  |  | Tachinidae sp. 5 |  |  | 3 |
| Spid.-hunt. | *Trypoxylon* sp. 2 |  | 7 |  |  | Braconidae sp. 2 | 1 | 1 |  |
| Spid.-hunt. | *Trypoxylon malaisei* | 5 |  |  |  | Ichneumonidae sp. 2 |  | 2 |  |
| Spid.-hunt. | *Auplopus* sp. 3 |  |  | 4 |  | Tachinidae sp. 1 | 1 | 1 |  |
| Spid.-hunt. | *Auplopus* sp. 5 |  |  | 4 |  | Braconidae sp. 3 |  | 2 |  |
| Spid.-hunt. | *Nitela* sp. 1 | 4 |  |  |  | *Comastichus zopheros* | 1 | 1 |  |
|  |  |  |  |  |  | *Copidosoma nacoleiae* |  | 2 |  |
|  |  |  |  |  |  | Phoridae sp. 1 |  |  | 2 |
|  |  |  |  |  |  | Sarcophagidae sp. 3 |  | 2 |  |
|  |  |  |  |  |  | Sarcophagidae sp. 5 |  | 2 |  |
|  |  |  |  |  |  | Tachinidae sp. 2 | 2 |  |  |
|  |  |  |  |  |  | *Macrosiagon nasutum* |  | 1 |  |
|  |  |  |  |  |  | Braconidae sp. 2 |  | 1 |  |
|  |  |  |  |  |  | *Chrysis lamellata* |  | 1 |  |
|  |  |  |  |  |  | *Coelioxys* sp. 3 | 1 |  |  |
|  |  |  |  |  |  | *Eurytoma* sp. 2 |  | 1 |  |
|  |  |  |  |  |  | Ichneumonidae sp. 10 | 1 |  |  |
|  |  |  |  |  |  | Ichneumonidae sp. 9 | 1 |  |  |
|  |  |  |  |  |  | *Coelioxys* sp. 5 |  | 1 |  |
|  |  |  |  |  |  | *Leucospis* sp. 2 | 1 |  |  |
|  |  |  |  |  |  | Mutillidae sp. 1 |  |  | 1 |
|  |  |  |  |  |  | *Omalus pseudoimbecillus* | 1 |  |  |
|  |  |  |  |  |  | *Pseudomalus joannis* | 1 |  |  |
|  |  |  |  |  |  | *Taeniogonalos fasciata* |  |  | 1 |

Supplementary Table 3:

Statistical results table of SEMs testing all direct and indirect correlations of all response variables and among response and predictor variables. – no correlations were identified; ~ correlation was expected, not directional and not tested; SE: standard error; Df: degrees of freedom. The effect of covariate ‘site’ was tested on each response; significant effects of the experimental young forest ‘site A’ in comparison to ‘site B’ are shown. A statistical alternative model for herbivore-hunting wasps replaces the path between canopy and wasp abundance with a path between basal area and wasp abundance (ΔAICc = 1, Estimate = 0.3293, SE = 0.0772, DF = 86, *P*-value < 0.0001, R^2^_abundance_ = 0.288). A statistical alternative model for the model including climate data replaces the negative correlation between canopy cover and temperature with a negative correlation between basal area and temperature (ΔAICc = 1, Estimate = -0.2685, SE = 0.0532, DF = 52, *P*-value < 0.0001, R^2^_temperature_ = 0.403)

| Response | Predictor | Estimate | *SE* | *Df* | *P*-value |
| --- | --- | --- | --- | --- | --- |
| **Host and natural enemies (full data)** | | | | | |
| Enemy species richness | Enemy abundance | 0.0283 | 0.0035 | 86 | 0.0000 |
| Enemy abundance | Host abundance | 0.0074 | 0.0019 | 85 | 0.0001 |
| Enemy abundance | Site | -0.3456 | 0.1465 | 85 | 0.0183 |
| Host species richness | - |  |  |  |  |
| Host abundance | Canopy cover | 0.5662 | 0.1846 | 85 | 0.0022 |
| Host abundance | Site | 0.3163 | 0.1287 | 85 | 0.0140 |
| Basal area | Site | 0.8065 | 0.1970 | 86 | 0.0001 |
| Canopy cover | Basal area | 1.4749 | 0.3433 | 86 | 0.0000 |
| **Host and natural enemies (subset of 55 plots with microclimate data)** | | | | | |
| Enemy species richness | Enemy abundance | 0.0242 | 0.0047 | 52 | 0.0000 |
| Enemy species richness | Temperature | -0.3508 | 0.1252 | 52 | 0.0051 |
| Enemy abundance | Relative humidity | 0.0988 | 0.0359 | 53 | 0.0060 |
| Host species richness | - |  |  |  |  |
| Host abundance | Relative humidity | 0.1061 | 0.0310 | 52 | 0.0006 |
| Host abundance | Site | 0.7657 | 0.1457 | 52 | 0.0000 |
| Temperature | Canopy cover | -0.8735 | 0.1511 | 52 | 0.0000 |
| Temperature | Site | 0.3141 | 0.1111 | 52 | 0.0067 |
| Relative humidity | Canopy cover | 3.2277 | 0.6923 | 52 | 0.0000 |
| Relative humidity | Site | -2.7557 | 0.5091 | 52 | 0.0000 |
| ~ Temperature | ~ relative humidity | -0.8383 | - | 55 |  |
| Canopy cover | Basal area | 1.3257 | 0.3825 | 53 | 0.0005 |
| Basal area | Site | 0.8157 | 0.2893 | 53 | 0.0068 |
| **Bees (full data)** |  |  |  |  |  |
| Enemy species richness | Enemy abundance | 0.1299 | 0.0154 | 79 | 0.0000 |
| Enemy abundance | Host abundance | 0.0122 | 0.0048 | 78 | 0.0104 |
| Enemy abundance | Basal area | 0.3561 | 0.132 | 78 | 0.0070 |
| Host species richness | Site | 0.4418 | 0.1479 | 86 | 0.0028 |
| Host abundance | Site | 0.6816 | 0.2074 | 86 | 0.0010 |
| Basal area | Site | 0.8065 | 0.197 | 86 | 0.0001 |
| Canopy cover | Basal area | 1.4749 | 0.3433 | 86 | 0.0000 |
| **Herbivore-hunting wasps full data)** | |  |  |  |  |
| Enemy species richness | Enemy abundance | 0.0476 | 0.0073 | 83 | 0.0000 |
| Enemy abundance | Host abundance | 0.0179 | 0.0022 | 82 | 0.0000 |
| Enemy abundance | Site | -0.4257 | 0.1439 | 82 | 0.0031 |
| Host species richness | Host abundance | 0.0043 | 0.0019 | 86 | 0.0238 |
| Host abundance | Canopy cover | 1.0381 | 0.2196 | 86 | 0.0000 |
| Basal area | Site | 0.8065 | 0.197 | 86 | 0.0001 |
| Canopy cover | Basal area | 1.4749 | 0.3433 | 86 | 0.0000 |
| **Spider-hunting wasps (full data)** | | |  |  |  |
| Enemy species richness | Enemy abundance | 0.0746 | 0.0142 | 71 | 0.0000 |
| Enemy abundance | Host abundance | 0.0717 | 0.0087 | 71 | 0.0000 |
| Host species richness | Host abundance | 0.0258 | 0.0066 | 86 | 0.0001 |
| Host abundance | Site | -0.3420 | 0.2178 | 86 | 0.1164 |
| Basal area | Site | 0.8065 | 0.197 | 86 | 0.0001 |
| Canopy cover | Basal area | 1.4749 | 0.3433 | 86 | 0.0000 |


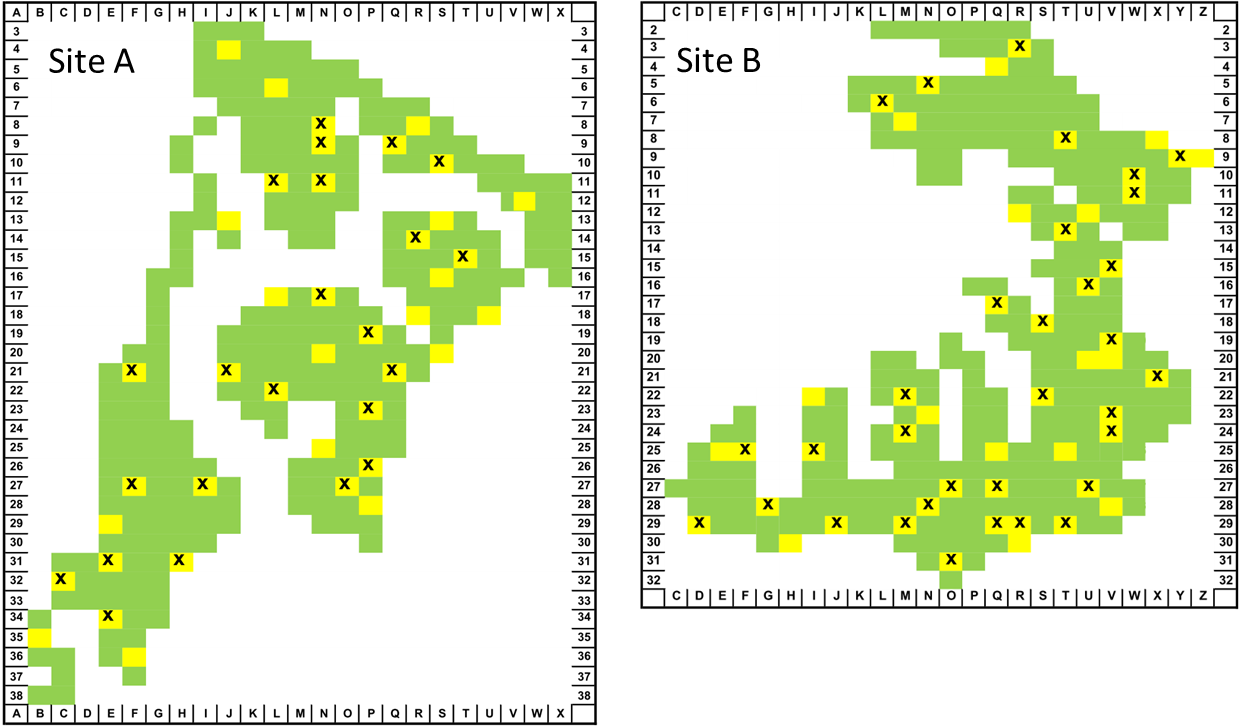


Supplementary Figure 1:

Locations of study plots within the two experimental young forest sites (A and B) of the BEF-China experiment. Study plots, highlighted in yellow, from which cavity-nesting Hymenoptera were collected, are randomly distributed across the sites, surrounded by further plots of the experimental forest in green. Plots with microclimate measurements are labeled with an x. Site A is 18.4 ha and site B 20 ha in size, both sites are approximately 4 km apart. Each plot is 25.8 x 25.8 m, two traps were installed along a SW-NE diagonal at the center of each plot, with 11 m distance between traps.


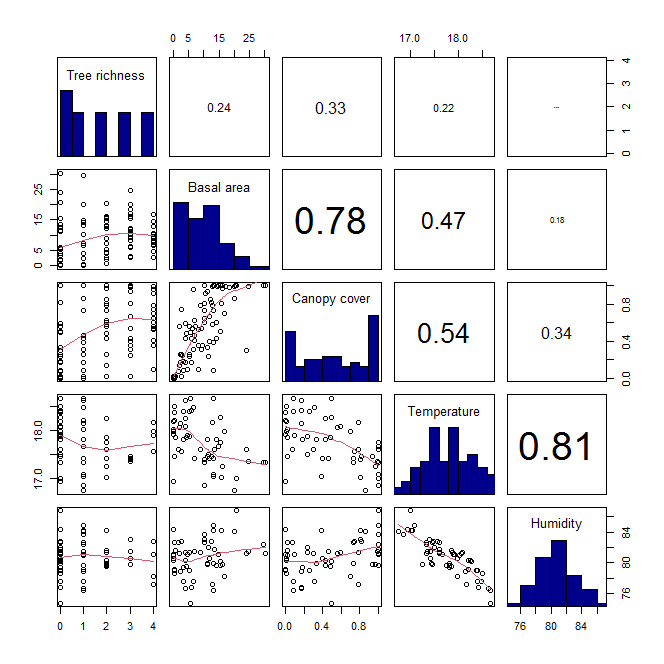
Supplementary Figure 2:

Data distribution and correlation coefficients between all pairs of predictor variables. Tree species richness values are log_2_ transformed. Lower panel shows scatter plots of individual data points and a smoother for each pair of predictors, diagonal panel shows histograms of data distribution for each predictor, upper-left panel shows spearman correlation coefficients between pairs of predictors (increasing font indicates increasing correlation between pairs).


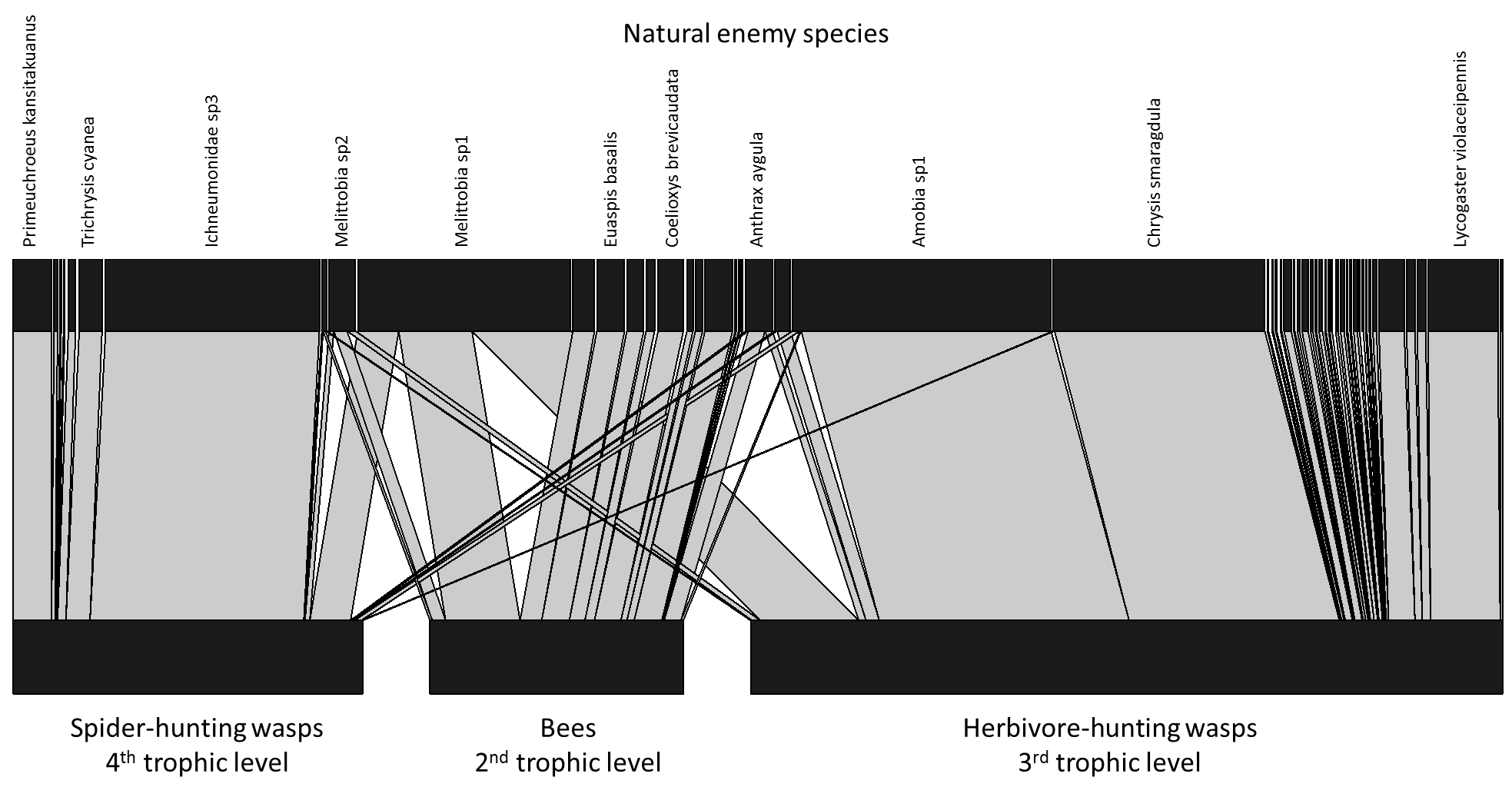


Supplementary Figure 3:

Bipartite interaction network between natural enemies (upper boxes) and hosts grouped by trophic position (lower boxes). Natural enemies form distinct clusters with few (7 out of 59) species attacking hosts at different trophic levels. In SEM analysis (Fig. 3), each natural enemy individual was assigned one trophic level higher than its host individual. Connecting links represent the frequency of interactions between natural enemy species and host groups. The underlying abundance of hosts is not shown and only common natural enemies are labeled (see Supplementary Table 2 for the full species list). The width of boxes and links represents the relative abundance and frequency of individuals or interactions. The graph was plotted with the bipartite package in R (Dormann et al. 2009).


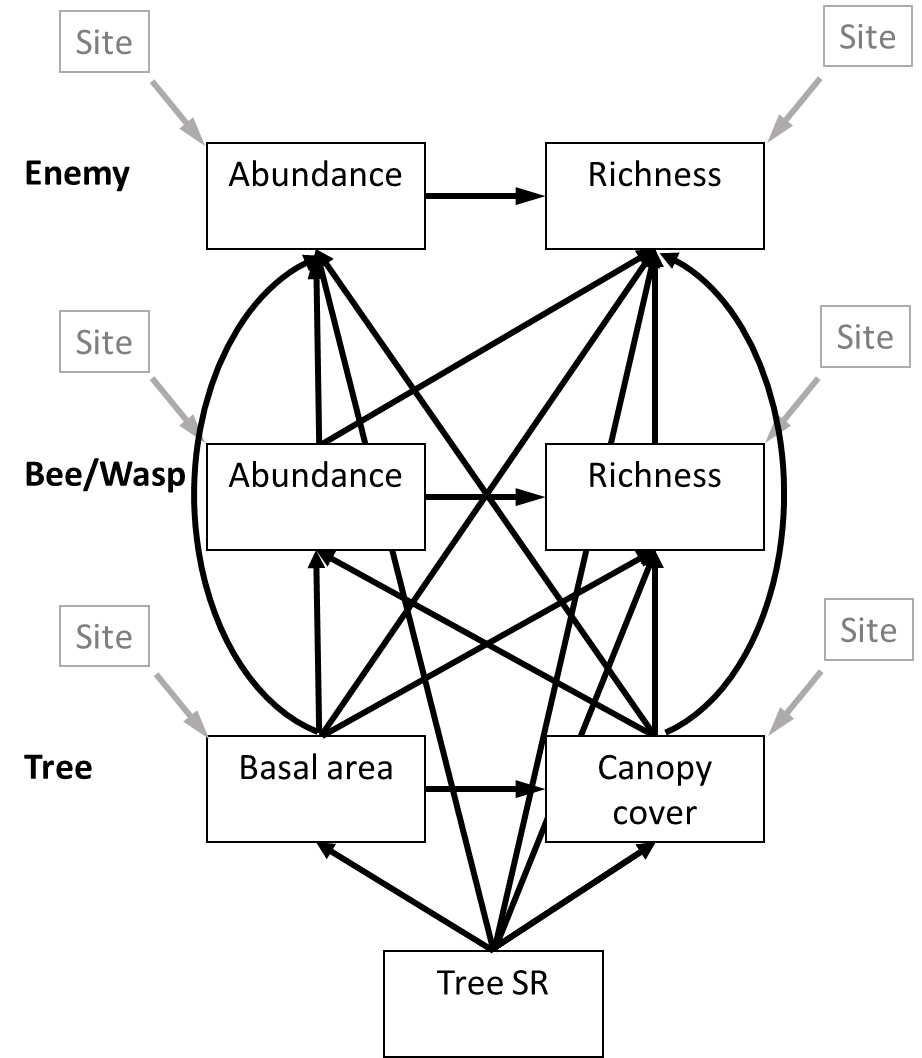


Supplementary Figure 4:

Example full SEM including all path from below to top and from left to right parameters (basal area to canopy cover and abundance to species richness) and the effect of site on all parameters. The same approach was used for the climate data model on the subset data and on SEMs for the individual host (bee and wasp) groups.


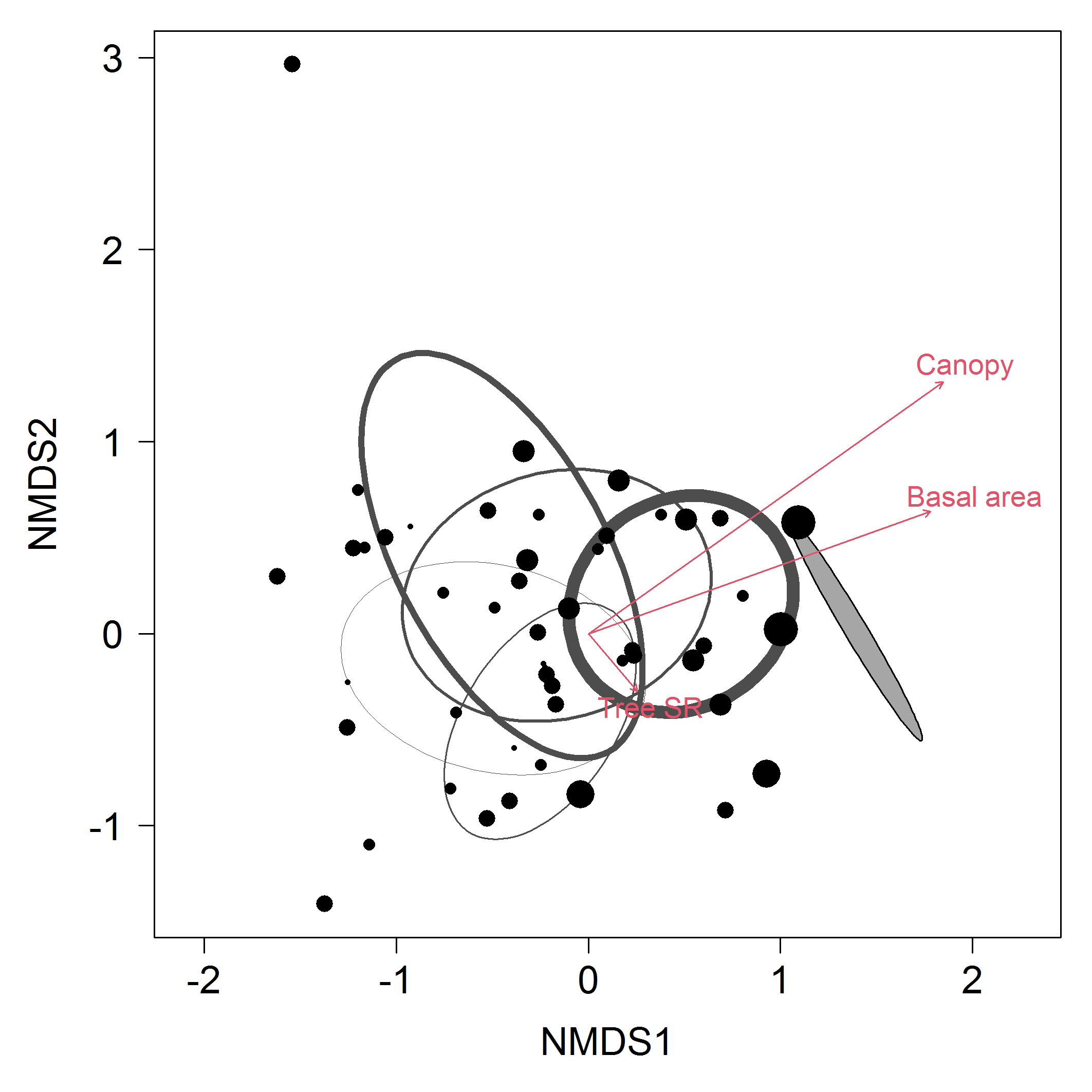

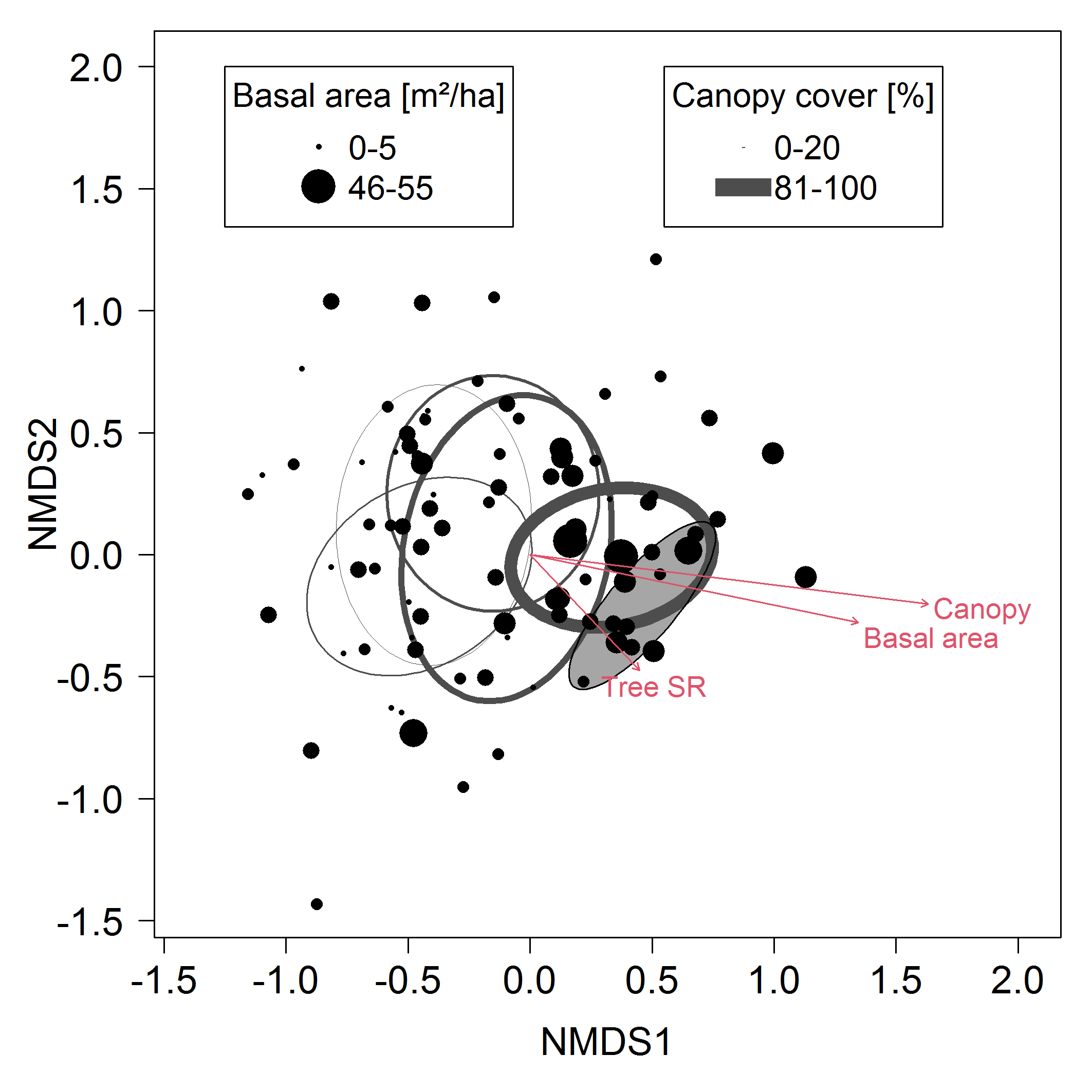

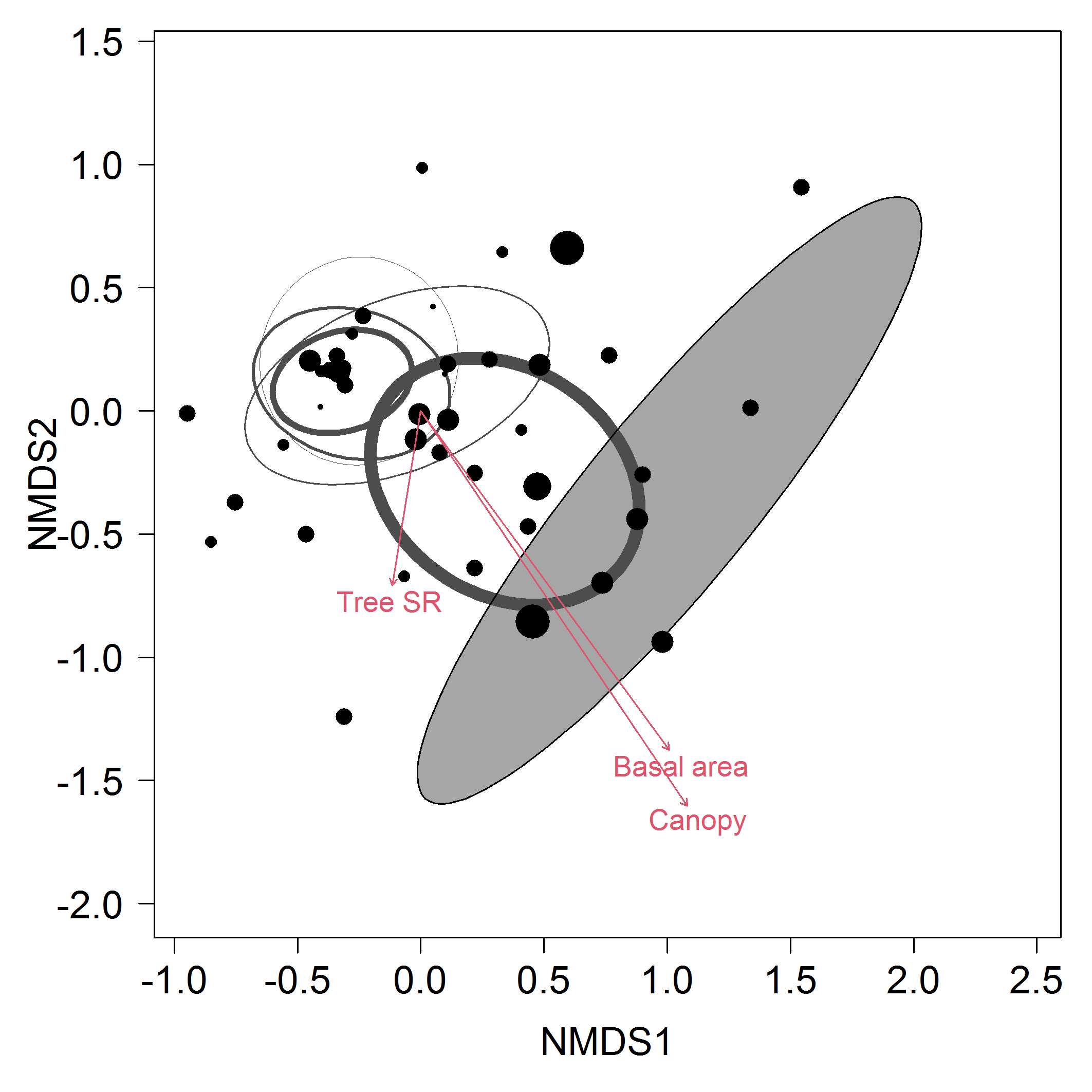


a

c

b

Supplementary Figure 5:

Non-metric multidimensional scaling (NMDS) showing the first two dimensions of ordinations of the cavity-nesting species community matrix for A) bees (stress = 0.105, k = 2), B) herbivore-hunting wasps (stress = 0.180, k = 3) and C) spider-hunting wasps (stress = 0.079, k = 3). Ellipses show 95% confidence intervals of communities grouped by canopy cover. With increasing line thickness (canopy cover) and point size (basal area) the bee and wasp communities changed. The shaded ellipse represents the respective insect communities of a nearby natural forest (CSPs) which were not used for environmental variable fitting. Red arrows indicate the post-hoc correlations with canopy cover, basal area and tree species richness (bees, basal area *P* < 0.001, canopy cover *P* < 0.001, tree richness *P* = 0.757; herbivore-hunting wasps, basal area *P* < 0.001, canopy cover *P* < 0.001, tree richness *P* = 0.120; spider-hunting wasps, basal area *P* < 0.001, canopy cover *P* < 0.001, tree richness *P* = 0.643).


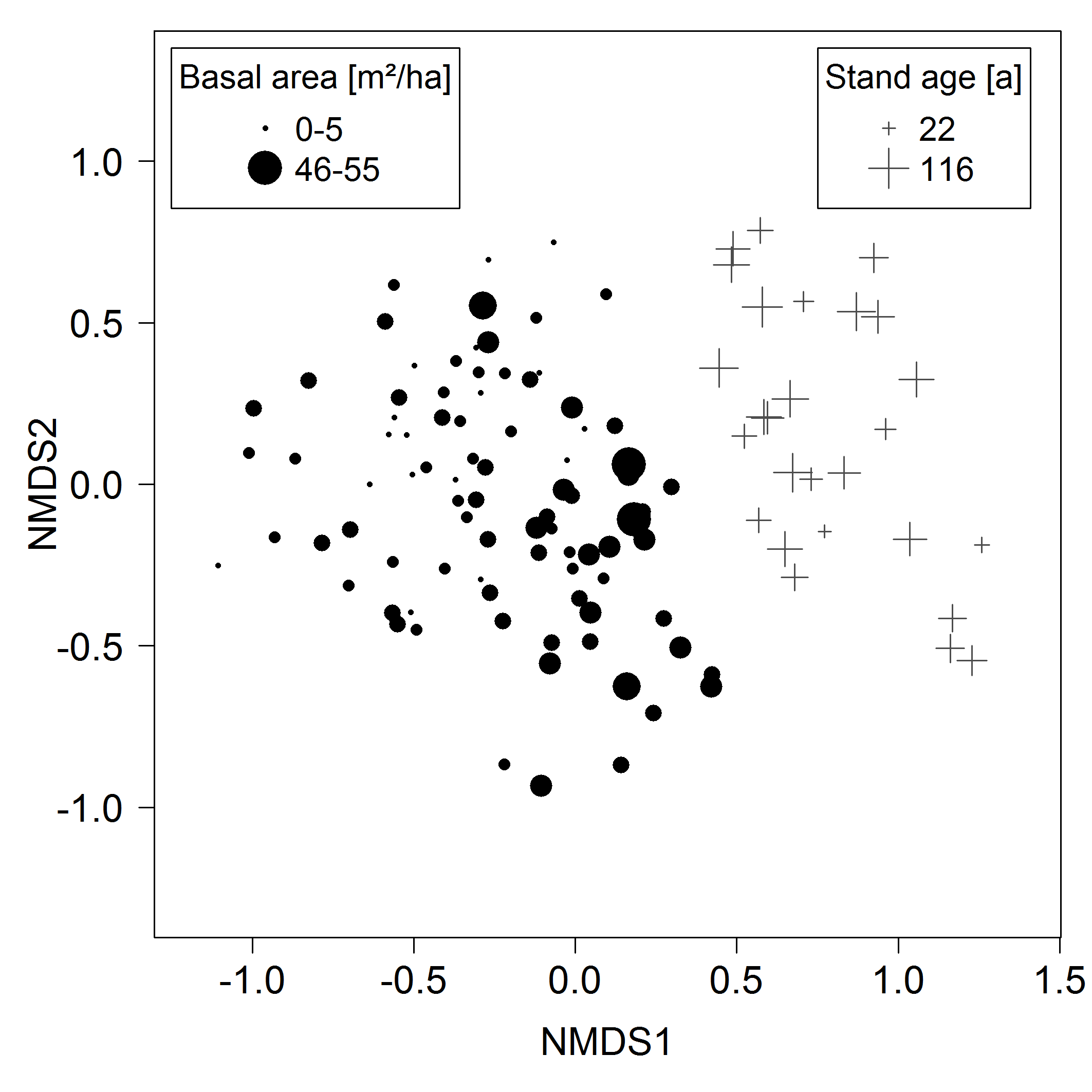


Supplementary Figure 6:

Replicate of Main Fig 1 but with visualization of the individual bee and wasp communities of the CSP natural forest plots (+), where increasing symbol size indicates the stand age of a plot. An analysis of the natural forest communities showed no correlation of reference community dissimilarity and stand age, ranging from plots with maximum 22 to plots with maximum 116 year old trees (stand age is the age of the fifth largest tree in a plot (Bruelheide et al. 2011)). The point size (dots) of the young, experimental forests corresponds to plot mean basal area.

Supplementary References:

Bruelheide H, Böhnke M, Both S, et al (2011) Community assembly during secondary forest succession in a Chinese subtropical forest. Ecol Monogr 81:25–41. https://doi.org/10.1890/09-2172.1

Dormann CF, Fründ J, Blüthgen N, Gruber B (2009) Indices, graphs and null models: analyzing bipartite ecological networks. Open Ecol J 2:7–24. https://doi.org/10.2174/1874213000902010007

Supplementary Data 1:

Predictor variables, canopy cover, basal area and tree species richness (SR) of all 88 experimental plots. Canopy cover represents the vegetation-covered area of hemispherical pictures taken at each trap position, basal area in m² ha^-1^, see Methods 2.4.

| **Site** | **Plot** | **Canopy cover** | **Basal area** | **Tree SR** |  | **Site** | **Plot** | **Canopy cover** | **Basal area** | **Tree SR** |
| --- | --- | --- | --- | --- | --- | --- | --- | --- | --- | --- |
| A | B35 | 0.58 | 21.17 | 4 |  | B | D29 | 0.00 | 0.00 | 1 |
| A | C32 | 1.00 | 49.06 | 2 |  | B | E25 | 0.92 | 17.66 | 16 |
| A | E29 | 0.98 | 30.78 | 8 |  | B | F25 | 0.35 | 13.94 | 8 |
| A | E31 | 0.26 | 8.90 | 1 |  | B | G28 | 1.00 | 17.02 | 2 |
| A | E34 | 1.00 | 34.62 | 1 |  | B | H30 | 0.56 | 9.06 | 4 |
| A | F21 | 0.08 | 5.34 | 1 |  | B | I22 | 0.65 | 16.97 | 16 |
| A | F27 | 1.00 | 25.90 | 4 |  | B | I25 | 0.23 | 18.97 | 1 |
| A | F36 | 0.09 | 13.80 | 16 |  | B | J29 | 0.40 | 20.02 | 8 |
| A | H31 | 1.00 | 24.89 | 2 |  | B | L6 | 0.36 | 15.91 | 16 |
| A | I27 | 0.08 | 21.68 | 2 |  | B | M22 | 0.79 | 14.59 | 4 |
| A | J13 | 0.85 | 20.28 | 4 |  | B | M24 | 0.73 | 16.87 | 2 |
| A | J21 | 0.46 | 11.84 | 2 |  | B | M29 | 0.43 | 23.80 | 2 |
| A | J4 | 0.41 | 11.52 | 16 |  | B | M7 | 0.59 | 6.50 | 1 |
| A | L11 | 0.33 | 18.76 | 1 |  | B | N23 | 0.43 | 10.15 | 8 |
| A | L17 | 1.00 | 26.84 | 8 |  | B | N28 | 0.01 | 0.41 | 1 |
| A | L22 | 0.70 | 27.84 | 16 |  | B | N5 | 0.56 | 3.31 | 1 |
| A | L6 | 0.56 | 12.38 | 16 |  | B | O27 | 0.39 | 21.82 | 2 |
| A | N11 | 0.50 | 9.19 | 1 |  | B | O31 | 0.81 | 26.34 | 4 |
| A | N17 | 0.19 | 5.40 | 1 |  | B | Q17 | 0.81 | 16.78 | 8 |
| A | N20 | 1.00 | 34.10 | 4 |  | B | Q25 | 0.54 | 15.33 | 16 |
| A | N25 | 0.99 | 33.39 | 8 |  | B | Q27 | 1.00 | 22.56 | 1 |
| A | N8 | 0.73 | 22.03 | 4 |  | B | Q29 | 0.08 | 2.83 | 1 |
| A | N9 | 0.79 | 21.15 | 16 |  | B | Q5 | 0.54 | 10.71 | 8 |
| A | O27 | 1.00 | 50.37 | 1 |  | B | R12 | 0.31 | 15.27 | 8 |
| A | P19 | 0.98 | 27.00 | 4 |  | B | R29 | 0.23 | 8.66 | 1 |
| A | P23 | 0.86 | 33.28 | 2 |  | B | R3 | 0.43 | 6.86 | 4 |
| A | P26 | 0.31 | 10.84 | 2 |  | B | R30 | 0.53 | 13.49 | 16 |
| A | P28 | 0.67 | 19.59 | 8 |  | B | S18 | 0.02 | 1.01 | 4 |
| A | Q21 | 0.17 | 6.43 | 2 |  | B | S22 | 0.09 | 7.60 | 16 |
| A | Q9 | 0.17 | 3.76 | 2 |  | B | T13 | 0.01 | 0.00 | 1 |
| A | R14 | 0.17 | 10.12 | 1 |  | B | T25 | 0.60 | 23.83 | 4 |
| A | R18 | 1.00 | 21.16 | 16 |  | B | T29 | 0.02 | 0.42 | 1 |
| A | R8 | 1.00 | 25.88 | 8 |  | B | T8 | 0.74 | 4.73 | 16 |
| A | S10 | 1.00 | 40.81 | 8 |  | B | U12 | 0.60 | 17.13 | 16 |
| A | S13 | 0.99 | 28.43 | 8 |  | B | U16 | 0.50 | 25.60 | 1 |
| A | S16 | 0.26 | 4.63 | 4 |  | B | U20 | 0.62 | 11.84 | 4 |
| A | S20 | 0.95 | 10.81 | 16 |  | B | U27 | 0.00 | 0.90 | 2 |
| A | T15 | 0.94 | 18.72 | 8 |  | B | V15 | 0.02 | 0.18 | 2 |
| A | U18 | 0.99 | 24.32 | 16 |  | B | V19 | 0.26 | 3.78 | 2 |
| A | W12 | 0.49 | 7.04 | 4 |  | B | V20 | 0.25 | 5.04 | 8 |
|  |  |  |  |  |  | B | V23 | 1.00 | 21.74 | 2 |
|  |  |  |  |  |  | B | V24 | 0.82 | 20.75 | 1 |
|  |  |  |  |  |  | B | V28 | 0.02 | 4.66 | 8 |
|  |  |  |  |  |  | B | W10 | 0.00 | 0.55 | 1 |
|  |  |  |  |  |  | B | W11 | 0.30 | 39.85 | 1 |
|  |  |  |  |  |  | B | X21 | 0.00 | 0.19 | 1 |
|  |  |  |  |  |  | B | X8 | 0.14 | 3.62 | 4 |
|  |  |  |  |  |  | B | Y9 | 0.01 | 0.00 | 1 |
